# Utility of Surgical Apgar Score in Predicting Post-operative Complications After Whipple Procedure in Pancreatic Cancer Patients

**DOI:** 10.1007/s13193-024-02151-1

**Published:** 2024-12-07

**Authors:** Nilanjana Howbora, Raghu S. Thota, Sagar Pargunde, Vijaya Patil, Vandana Agarwal, Manish Bhandare, Shailesh V. Shrikhande

**Affiliations:** 1 Dr. B. Borooah Cancer Institute, Guwahati, Assam India; 2 Tata Memorial Centre, Mumbai, India; 3 Dr. Vaishampayan Memorial Govt Medical College, Solapur, Maharashtra India

**Keywords:** Surgical Apgar Score, Pancreatic cancer, Whipple procedure, Post-operative complications

## Abstract

An intraoperative 10-point Surgical Apgar Score, based on estimated blood loss, lowest mean arterial pressure, and lowest heart rate, was developed and validated for predicting postoperative complications in patients undergoing vascular and general surgeries. We sought to estimate the ability of this metric to predict major postoperative complications in patients with pancreatic cancer undergoing the Whipple procedure. This is a prospective, observational, single-centre study involving adult patients undergoing the Whipple procedure, at a major tertiary cancer centre. All eligible patients undergoing Whipple surgery in our institute between March 2018 and October 2021 were included in the study. Demographic data, clinicopathological characteristics, comorbidities, intraoperative variables, and postoperative complications were analyzed. The surgical score was calculated from intraoperative blood loss, lowest heart rate, and lowest mean arterial pressure. All the patients were followed up till 30 days postoperatively. Descriptive statistics and univariate and multivariate analyses were used as appropriate. The occurrence of major postoperative complications represented the primary outcome. A total of 253 patients were analyzed. The mean duration of surgery was 436 min. On statistical analyses, the occurrence of major postoperative complications was significantly associated with SAS ≤ 4 (OR = 8.00, 95% CI = 3.78–16.93, *p* = 0.000), use of intraoperative vasopressor (OR = 2.247, 95% CI = 1.312–3.846, *p* = 0.003), and body mass index (BMI) (OR = 1.074, 95% CI = 1.010–1.142, *p* = 0.022). However, we did not find any significant association between other demographic variables like age, comorbidities, duration of surgery, and preoperative s. albumin with the occurrence of postoperative complications. Lower SAS (≤ 4) is the most powerful predictor of postoperative complications in pancreatic cancer patients undergoing Whipple surgery. The score provides a simple and immediate means of measuring and communicating patient outcomes, using data routinely available in any setting.

## Introduction

Whipple procedure is the surgical treatment of choice for pancreatic cancer. The 5-year survival rate for patients improves from 7.7% to about 20% in those undergoing the surgery [1, 2]. However, the pancreaticoduodenectomy itself carries a significant risk of complications (30–60%), with a duration of ICU monitoring and hospital stay being longer than that for most other upper gastrointestinal surgeries [3]. Although many of the complications respond to medical treatment or radiological/endoscopic interventions, complications that require redo surgeries, carry a mortality risk of 23–67% [4].

The possibility of identifying those patients who are at risk for postoperative complications and targeting them for early treatment gives an opportunity to develop interventions that might significantly improve outcomes of the surgeries. Scores such as ASA (American Society of Anaesthesiologist) grading [5], POSSUM (Physiological and Operative Severity Score for the enUmeration of Mortality and Morbidity) [21], APACHE II (Acute Physiology and Chronic Health Evaluation II) [20] are accurate but difficult to adopt into routine clinical practice owing to their complex nature. The Surgical Apgar Score (SAS) developed by Gawande et al. [6] take into account intraoperative blood loss, lowest blood pressure, and lowest heart rate, and a lower score on a scale of 0 to 10 predicts a poorer prognosis (Table 1). This is easy to understand and implement and has shown a strong correlation with the occurrence of major complications/death within 30 days of surgery [7] and has since been validated in general, vascular, urological, gynaecological, orthopaedic, and neurosurgery [8–10].

**Table 1:** Surgical Apgar Score

Surgical Apgar Score points					
	0 point	1 point	2 points	3 points	4 points
Estimated blood loss (ml)	> 1000	600–1000	101–600	≤ 100	-
Lowest mean arterial pressure (mm Hg)	< 40	40–54	55–69	≥ 70	-
Lowest heart rate (beats/min)	> 85	76–85	66–75	56–65	≤ 55

The purpose of our study was to assess if SAS could predict major postoperative complications among patients undergoing the Whipple procedure in patients with pancreatic cancer.

## Methodology

### Study Setting

This was a single-centre, prospective, observational study, carried out after Institutional Ethics Committee (IEC) and Clinical Trial Registry of India (CTRI) approval at a tertiary care oncology centre in Western India. The study included patients undergoing the Whipple procedure for pancreatic cancer, between the age group of 18–75 years, with ASA-PS I–III, between March 2018 and October 2021. Patients were recruited 1 day prior to the surgery after taking a written informed consent. Preoperative demographic characteristics and detailed medical and surgical history were recorded for all participants. Refusal to give consent was the only criterion for exclusion.

### Study Procedures

Administration and conduct of anaesthesia, including fluid management, analgesia, and reversal of neuromuscular blocking drugs, were carried out as per the standard institutional practice, at the discretion of anaesthesiologist in charge. Pre-operative demographic characteristics such as age, sex, height, weight, BMI, and details of patient’s medical (presence of comorbidities, ongoing treatment, preoperative chemotherapy, preoperative S. albumin levels) and surgical history were noted. The intraoperative haemodynamic data required to calculate the Surgical Apgar Score (SAS) was acquired for all the patients. Data on the duration of the surgery and the use of vasopressors during the surgery was also recorded. SAS was calculated from 3 intraoperative variables: the estimated blood loss (EBL), the lowest heart rate (HR), and the lowest mean arterial pressure (MAP). For the purpose of analysis, SAS was grouped as low (≤ 4 points), intermediate (5–6 points), and high (7–10 points). Post-operative complications including mortality were assessed up to 30 days by interviews during follow-up visits or via phone calls for those unable to follow up physically. These complications were categorized as major and minor, which is consistent with NSQIP definitions (Table 2).

**Table 2:** Major and minor complications

Major complications	Minor complications
1. Acute kidney injury	1. Urinary tract infections
2. Blood loss of ≥ 2000 ml, transfusion requiring ≥ 4 U-packed blood cells within 72 h of surgery	2. Nausea or vomiting
3. Cardiac or respiratory arrest needing cardiopulmonary resuscitation (CPR) or coma for 24 h or more	3. Dehydration
4. Deep venous thrombosis	4. Fever above 101.5 F greater than 48 h postoperatively and
5. Septic shock	5. Superficial wound infection
6. Acute myocardial infarction	
7. New onset arrhythmias	
8. Unplanned re-intubation	
9. Ventilator use for 48 h or longer	
10. Pneumonia	
11. Pulmonary embolism	
12. Stroke, wound disruption	
13. Deep or organ space surgical site infection	
14. Sepsis	
15. Systemic inflammatory response syndrome	
16. Unplanned intensive care unit (ICU) admission	
17. Need for reoperation	
18. Postoperative ileus requiring TPN	
19. Anastomotic leak or fistula, vascular, ureteral, or neural injuries	
20. Unplanned readmission < 30 days of discharge	
21. Death	

### Outcome Measures

The occurrence of major postoperative complications and/or death within 30 days of surgery was the primary outcome of interest.

### Statistical Analysis

Statistical analysis was performed using SPSS (the statistical package for social sciences) version 24.0 (SPSS, Inc., Chicago, IL, USA). For continuous variables, mean and standard deviations are presented; for categorical variables, frequency and percentage values are presented. Chi-square test and Fischer’s exact test were used to compare categorical variables. To compare the two groups, Student’s *t* test was used for parametric conditions, and the Mann–Whitney *U* test was used for non-parametric conditions. Predictor factors of PORC were determined through univariate logistic regression. All parameters with *p* < 0.05 in univariate analyses were included in a multivariate logistic regression model. A *p*-value of ≤ 0.05 was considered statistically significant. The odds ratio of factors associated with PORC will be calculated with 95% confidence intervals.

## Results

All 265 patients who were scheduled to undergo a Whipple’s procedure consented to be a part of the study. Of these 265, 12 turned inoperable {in view of (a) metastasis to liver and peritoneum, (b) encasement of celiac artery, superior mesenteric artery by the tumour}, thus excluded from the study. The remaining 253 patients were studied and analyzed. There was no loss of data, nor any violation of protocols during the whole duration of the study, and all 253 patients included in the study were adequately followed up till 30 days postoperatively.

The demographics and pre/intraoperative variables are recorded in Table 3.

**Table 3:** Demographic and pre/intraoperative variables

Parameter	Mean (SD)
1. Age at presentation (years)	54 (11.16)
2. BMI (kg/m^2^)	23 (4.76)
3. Preop s. albumin (g/dl)	3.8(1.99)
4. Duration of surgery (min)	436 (125.28)
5. Lowest heart rate (per minute)	60
6. Blood loss (ml)	1111

One hundred twelve out of 253 (44.27%) patients had no comorbidities, 91 (35.97%) patients had 1 comorbidity, and 50 (19.76%) patients had ≥ 2 comorbidities. Only 30 patients among the total of 253 patients received preoperative/adjuvant chemotherapy, while 223 patients were posted for upfront surgery.

All 253 patients were followed up for 30 days postoperatively for the occurrence of any complications as defined earlier. It was found that 121 (47.8%) patients suffered from ≥ 1 major complication, while the rest 132 (52.1%) patients had an uneventful recovery with no complications within 30 days postoperatively. There were 6 cases of deaths recorded within 30 days of surgery. The postoperative complications seen in the patients and their frequency are depicted in Table 4.

**Table 4:** Frequencies of complications

Postoperative complications	*N*	%
Need for reoperation (including interventional radiology procedures)	80	31.62%
Deep or organ space surgical site infection	39	15.4%
Anastomotic leak or fistula	38	15.01%
Postoperative ileus requiring TPN	33	13.04%
Wound disruption	32	12.64%
Unplanned ICU admission	23	9.09%
Sepsis	17	6.71%
Pneumonia	10	3.95%
Unplanned reintubation	7	2.76%
Death	6	2.37%
DVT	6	2.37%
Acute kidney injury	4	1.58%
Acute myocardial infarction	3	1.185%
New onset sustained arrhythmias	3	1.185%
SIRS	3	1.185%
Ventilator use for 48 h or longer	2	0.79%
Cardio-respiratory arrest needing CPR	2	0.79%
Septic shock	2	0.79%
Pulmonary embolism	1	0.39%
Stroke	1	0.39%

Patient demographics and clinicopathologic characteristics are recorded in Table 5.

**Table 5:** Patient demographics and clinicopathologic characteristics

Variables	Complications					
	Yes (*N* = 121)			No (*N* = 132)		
	Mean	Median	SD	Mean	Median	SD
Age (years)	55.50	56.00	9.85	53.36	55.00	12.48
BMI (kg/m^2^)	23.90	23.12	5.28	22.56	21.95	4.24
Preoperative ALBUMIN (g/dl)	4.11	3.90	2.47	3.66	3.90	1.52
Duration of surgery (min)	451.20	450.00	116.38	447.58	420.00	135.27

**Fig. 1:**
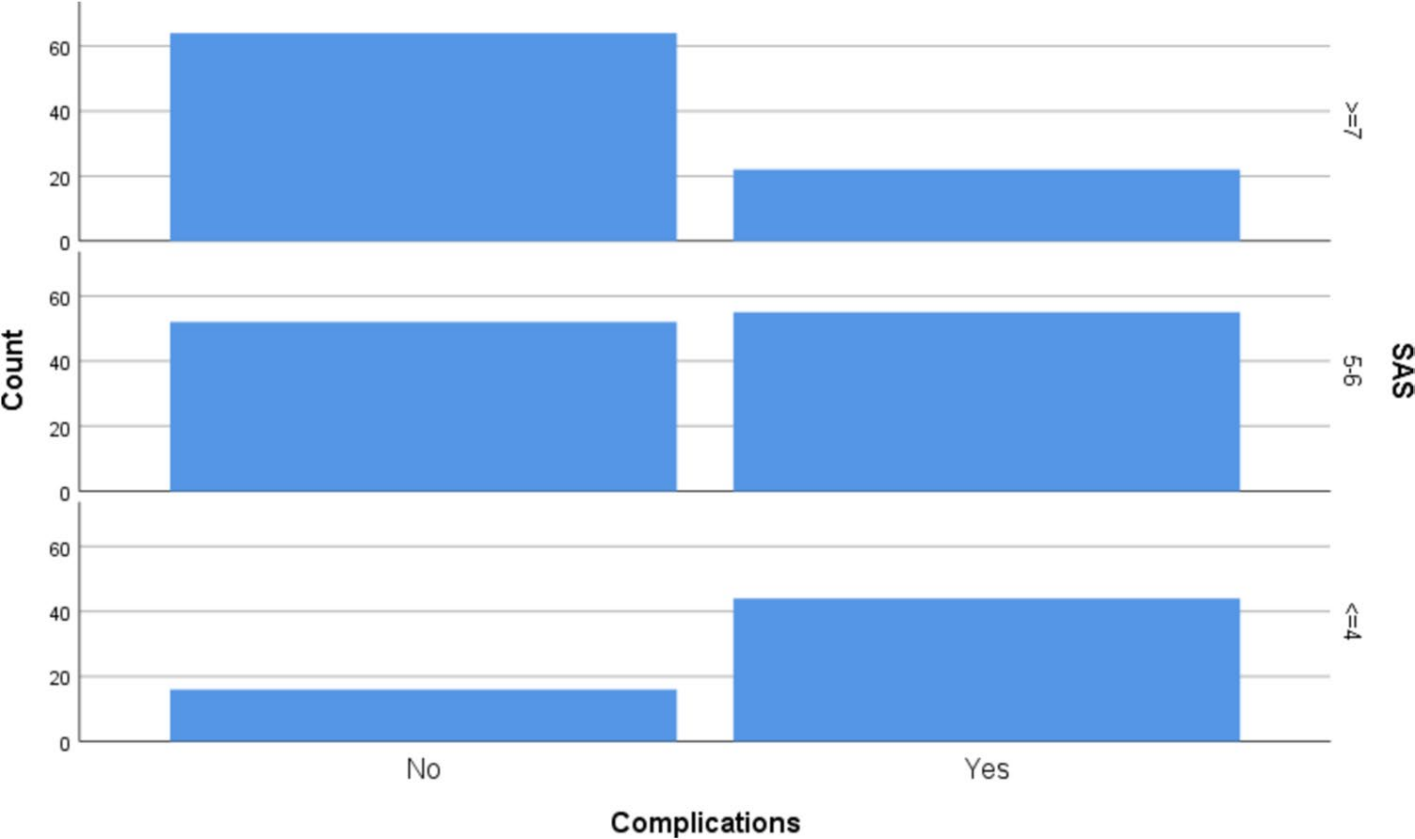
Graph showing the frequency of occurrence of postoperative complications in various categories of SAS

SAS was calculated for the entire cohort using intraoperative blood loss, lowest mean arterial pressure, and lowest heart rate as previously described. SAS was determined by adding points assigned for each of the 3 individual variables (Table 6) (Fig. 1).

**Table 6:** Frequency of occurrence of complications in various categories of SAS

Surgical Apgar Score (SAS)		
SAS categories	Complications	
	Yes (*n* = 121)	No (*n* = 132)
≤ 4 (*n* = 60)	44 (73.30%)	16 (26.70%)
5–6 (*n* = 107)	55 (51.40%)	52 (48.60%)
≥ 7 (*n* = 86)	22 (25.60%)	64 (74.40%)

Median SAS was 6 points. Forty-four out of 60 patients (i.e. 73.30%) with SAS of 1–4 were found to have ≥ 1 of the major postoperative complications described earlier. Among 107 patients who had a SAS of 5–6, 55 patients (i.e. 51.40%) had ≥ 1 complication, while out of 86 patients who had a SAS ≥ 7, only 22 patients (i.e. only 25.60%) had ≥ 1 postoperative complication.

**Fig. 2:**
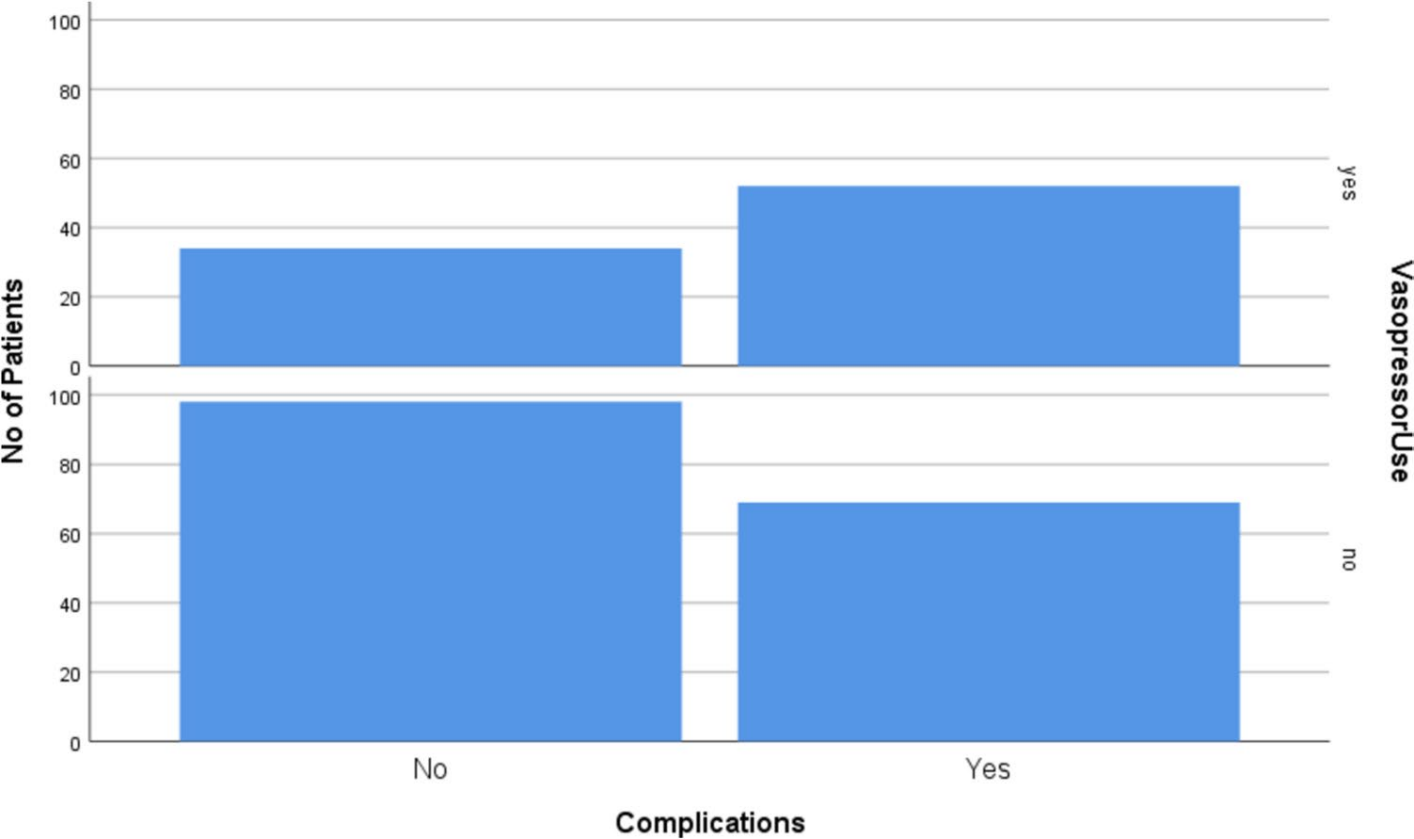
Graph showing the frequency of occurrence of postoperative complications with the use of vasopressors intraoperatively

Vasopressors were administered in 86 patients intraoperatively, among which 52 patients (i.e. 60.50%) had 1 or more postoperative complications, while only 69 (41.30%) among the 167 patients in whom no vasopressors were required intraoperatively suffered from postoperative complications (Table 7) (Fig. 2).

**Table 7:** Use of vasopressors and occurrence of postoperative complications

Use of intraoperative vasopressors		
Vasopressor use	Complications	
	Yes (*n* = 121)	No (*n* = 132)
Yes (*n* = 86)	52 (60.50%)	34 (39.50%)
No (*n* = 167)	69 (41.30%)	98 (58.70%)

On univariate analysis, significant postoperative complications were found to be associated with only the following components:

1. SAS ≤ 4 (*p* = 0.000) and SAS 5–6 (*p* = 0.000)
2. Use of intraoperative vasopressors (*p* = 0.003)
3. BMI (*p* = 0.022)

The results of multivariate analysis are depicted in Table 8.

**Table 8:** Results of multivariate analysis

Variables in the equation									
		*B*	S.E	Wald	df	Sig	Odds ratio	95% C.I. for EXP(B)	
								Lower	Upper
Step 1^a^	SAS	− .551	.104	27.839	1	**.000**	**.577**	.470	.707
	Age	.021	.013	2.463	1	.117	1.021	.995	1.048
	Sex (1)	.527	.306	2.965	1	.085	1.694	.930	3.087
	BMI in kg/m^2^	.081	.033	5.969	1	**.015**	**1.084**	1.016	1.157
	Co-morbidities	− .002	.065	.001	1	.980	.998	.879	1.134
	Preop chemotherapy (1)	− .171	.432	.157	1	.692	.843	.361	1.965
	Albumin g/dl	.122	.118	1.067	1	.302	1.130	.896	1.425
	Duration of surgery	− .003	.001	7.030	1	**.008**	**.997**	.994	.999
	Vasopressor use (1)	.204	.331	.380	1	.538	1.227	.641	2.349
	Transfusion (1)	− 18.929	40192.811	.000	1	1.000	.000	.000	
	Lowest temperature	.009	.097	.009	1	.925	1.009	.835	1.220
	Constant	19.130	40192.811	.000	1	1.000	203163719.185		

^a^Variable(s) entered on step 1: SAS score, age, sex, BMI in kg/m^2^, comorbidities, preop chemo, Sr. albumin g/dl, duration of surgery, vasopressor use, transfusion, lowest temperature recorded

Data in bold emphasis indicates statistically significant factors

Out of 141 patients who had ≥ 1 comorbidity, 72 patients (i.e. 51.0%) had 1 or more complications, while 49 patients (i.e. 43.75%) out of 112 patients who had no preoperative comorbidities suffered from 1 or more of the postoperative complications (Table 9). In spite of the difference in the percentage of occurrence of complications between the 2 groups, the association between the presence of comorbidities and the occurrence of postoperative complications was not found to be significant.

**Table 9:** Frequency of complications in the presence and absence of comorbidities

Presence of comorbidities		
No of comorbidities	Complications	
	Yes (*n* = 121)	No (*n* = 132)
No comorbidities (*n* = 112)	49 (43.80%)	63 (56.30%)
1 comorbidity (*n* = 91)	44 (48.40%)	47 (51.60%)
≥ 2 comorbidities (*n* = 50)	28 (56.00%)	22 (44.00%)

Similarly, we failed to find any significant association between age, administration of adjuvant/preoperative chemotherapy, preoperative s. albumin levels, and duration of surgery with the occurrence of major postoperative complications.

On applying logistic regression on all the above variables, the results obtained are shown in Table 10.

**Table 10:** Association between postoperative complications and other variables

Variable		OR	*p*-value	CI
SAS	≤ 4	8.000	0.000	3.78–16.93
	5–6	3.077	0.000	1.66–5.69
	≥ 7		0.504	
Age		1.013	0.280	0.989–1.038
BMI		1.074	0.022	1.010–1.142
Preoperative albumin		1.108	0.248	0.931–1.317
Duration of surgery		1.000	0.931	0.998–1.002
Comorbidities		1.009	0.887	0.892–1.141
Preop chemotherapy		0.694	0.367	0.314–1.533
Use of intraoperative vasopressors		2.247	0.003	1.312–3.846

After controlling confounders, SAS ≤ 4 points was found to be the most significant predictor of major postoperative complications (OR = 8.000, 95% CI = 3.78–16.93, *p* = 0.000).

## Discussion

In our study, we found that the previously validated Surgical Apgar Score also predicts major postoperative complications in patients undergoing the Whipple procedure.

In the original development of the score, Gawande et al. [6] found that the SAS was associated with major surgical complications and death within 30 days for a cohort of general and vascular surgery patients with similar results in a much larger validation cohort at a different institution [7]. Other studies have demonstrated the ability of the SAS to predict outcomes in a wide range of international settings [11] and after a variety of surgical procedures with a few studies in pancreatic cancer patients [12].

We have found that the simple 10-point Surgical Apgar Score remains a useful measure of the patients’ risk of major complications, not only in the immediate postoperative period, but also up to 30 days postoperatively.

A SAS of ≤ 4 was found to be the most significant predictor of postoperative complications. Out of 86 patients who had a SAS of ≥ 7, only 22 patients (i.e. only 25.60%) had incidence of postoperative complications. In contrast, 44 out of 60 patients (i.e. 73.30%) with SAS of 1–4 were found to have ≥ 1 of the major postoperative complications. Despite a relatively low prevalence of scores of ≤ 4 (23.7%), the consistent trend toward worse outcomes suggests that the score has good discriminative ability across the full spectrum.

Unlike many of the previous studies, like the one done by Zighelboim et al. [9], on patients of advanced ovarian cancer, undergoing cytoreductive surgery, which showed that the postoperative outcomes not only depend on the intraoperative variables, but are also significantly associated with patient’s preoperative condition like age, sex, multiple comorbidities, and stage of the disease, our study failed to show any significant association between patients’ prior preoperative conditions like age, comorbidities, preoperative or adjuvant chemotherapy, and preoperative s. albumin levels with the outcome of postoperative complications.

Preoperative hypoalbuminemia has been consistently shown to be associated with postoperative complications [13–15]. Unlike Marco LaTorreet al. [12] who in their study had shown that preoperative hypoalbuminemia is significantly associated with postoperative complications, our study did not show any significant association.

Although we found that > 55% of patients with > 1 comorbidity had 1 or more than 1 postoperative complication, a significant association between the presence of preoperative comorbidities and the occurrence of postoperative complications could not be established.

Intraoperatively, besides the SAS, the results of our study showed that the use of vasopressors was one of the factors that showed a significant association with the postoperative occurrence of major complications. About 35% of the patients were started on vasopressors and 60% of these patients developed postoperative complications. Though this variable had a statistically significant association with postoperative morbidity, we should note that these patients were administered vasopressors for hypotension, which is one of the variables of SAS. This factor has not been studied in any earlier studies. Other intraoperative variables, e.g. the duration of surgery, did not affect the postoperative outcome.

Haemodynamic stability [16–18] and the amount of blood loss [19] during surgery have long been recognized as important independent factors in patient outcomes. SAS recognizes the collective importance of these variables and their potential contribution to an easily implemented intraoperative performance metric.

Routine surveillance of patients with low surgical scores (e.g. a score of 4 or less), even when no complications results, may help in early identification of latent safety problems.

## Summary and Conclusion

In summary, our results demonstrate that:

1. A simple Surgical Apgar Score (SAS) can be derived from intraoperative data alone. This 10-point score based on the lowest HR, lowest MAP, and EBL discriminates well between groups of patients at higher- and lower-than-average risk of major complications and death within 30 days of surgery.
2. The Surgical Apgar Score provides a measure of immediate postoperative condition and prognostication and therefore assists in the triage of patients following the Whipple procedure to optimal postoperative levels of care and facilitates management in the postoperative period.
3. As a decision support tool, the score can inform postoperative prognostication, communication, and triage, and as a simple intraoperative outcome measure tool, it may prove useful as an indicator of surgical performance.

## Data Availability

Not applicable.
